# Impact of Compression Force on Mechanical, Textural, Release and Chewing Perception Properties of Compressible Medicated Chewing Gums

**DOI:** 10.3390/pharmaceutics13111808

**Published:** 2021-10-29

**Authors:** Yuliia Maslii, Tetiana Kolisnyk, Olena Ruban, Olga Yevtifieieva, Svitlana Gureyeva, Andriy Goy, Giedre Kasparaviciene, Zenona Kalveniene, Jurga Bernatoniene

**Affiliations:** 1 Department of Industrial Technology of Drugs, National University of Pharmacy, 61002 Kharkiv, Ukraine; julia.masliy@gmail.com (Y.M.); kolisnyktatyana@gmail.com (T.K.); ruban_elen@ukr.net (O.R.); 2 Department of Pharmaceutical Chemistry, National University of Pharmacy, 61002 Kharkiv, Ukraine; jevt.23@gmail.com; 3 JSC Farmak, 04080 Kyiv, Ukraine; s.gureeva@farmak.ua (S.G.); a.goy@farmak.ua (A.G.); 4 Department of Drug Technology and Social Pharmacy, Lithuanian University of Health Sciences, LT-50161 Kaunas, Lithuania; Giedre.Kasparaviciene@lsmuni.lt (G.K.); Zenona.Kalveniene@lsmuni.lt (Z.K.)

**Keywords:** compressible medicated chewing gums, compression force, mechanical resistance, textural analysis, release profile, chewing perception properties, lysozyme hydrochloride, ascorbic acid

## Abstract

Medicated chewing gums (MCGs) represent a beneficial platform for realizing drugs intended for dental prophylaxis and treatment. The present study aimed to investigate the impact of compression force on the mechanical, textural, release, and chewing perception characteristics of compressible MCGs with the combination of lysozyme hydrochloride (LH) and ascorbic acid (AsA). Four batches of MCGs were obtained on a laboratory single-punch tablet machine applying different forces, i.e., 5, 7, 10, and 15 kN, and evaluated by their geometrical parameters, mechanical resistance, surface and internal structure characteristics, texture profile, release behavior, and perception attributes during mastication. It was found that increasing compression force slightly affected resistance to crushing and friability of MCGs, but resulted in surface smoothing and formation of a thicker layer with highly compacted particle arrangement. According to the texture analysis, increasing compression force led to harder and more adhesive gums, indicating possible difficulties in chewing and, therefore, impairment of their consumer properties. Lower compression forces were also found to be preferable in terms of better drug release from the obtained chewing gums. The volunteers’ assessment showed that an increase of compression force led to significantly raising the initial hardness and crumbliness as well as to decreasing the rate of the integral gum mass formation during mastication, which may negatively affect perceptive sensations when using MCGs. Based on the results obtained, the optimal compressing force was selected to be 7 kN, which allows obtaining MCGs with good organoleptic, mechanical, textural, and release properties.

## 1. Introduction

In recent years, the pharmaceutical research field has shown a growing interest towards patient-friendly drug delivery systems. One of those is medicated chewing gum (MCG). Considering MCG as a platform for drug delivery, it is clearly evident that the main feature of its administration is relatively long mastication. On the one hand, this gives valuable benefits: the drugs are well tolerated by patients and easy to use, as they do not require swallowing and using water. Moreover, the act of chewing itself has been found to relieve stress [1,2]. Besides, chewing serves as a powerful aid to intensify the release of the drug from the dosage unit. On the other hand, long residence in the oral cavity involves taste issues, and since most active ingredients possess bitterness, it limits the range of possible candidates for MCG incorporation. Nevertheless, even taste problems can be successfully solved via certain technological approaches as was demonstrated in several works regarding MCG formulation studies [3,4].

Despite many applications which have been proposed, the feature of continuous chewing makes MCGs a prime candidate for use in dental prophylaxis and treatment. The potential of MCGs for the delivery of oral therapeutics is widely discussed in the literature. The chewing process is considered to have a positive effect on oral healthcare through facilitating the removal of sugars and bacterial fermentation end products, increasing the buffering action of saliva, enhancing the degree of remineralization of teeth, etc. [5,6,7,8].

It should be noted that in the case of compressible MCGs, this dosage form can be regarded as not only patient-friendly but also manufacturing friendly, which, unlike other innovative forms, can be easily implemented on a pharmaceutical scale without the need for highly specialized equipment [9,10,11]. Indeed, there are several brands of directly compressible gum bases currently available in the pharmaceutical excipient market that allow obtaining MCGs by a simple compression method using common tableting machines. Among such compressible gum bases, Health-in-Gum^®^ produced by Cafosa (Spain) appears to be the most mentioned in the literature [11,12,13].

For now, MCGs are a pharmacopoeial dosage form included in both the European Pharmacopoeia (Ph. Eur.) and the United States Pharmacopeia (USP) [14,15]. According to Ph. Eur. requirements, MCGs should meet practically the same standards as conventional tablets. The mandatory tests for MCGs are “Uniformity of dosage units” (2.9.40), “Uniformity of content” (2.9.6), “Uniformity of mass” (2.9.5), “Dissolution test for medicated chewing gums” (2.9.25), and “Microbiological quality of non-sterile pharmaceutical preparations and substances for pharmaceutical use” (5.1.4). However, currently, there are no official requirements on the mechanical (textural) properties of MCGs, while these characteristics are responsible for the chewiness and thus would affect the release behavior, as well as patient adherence and compliance to the treatment. In view of this, several techniques have been proposed for mechanical testing of MCGs, and texture profile analysis is among them [10,12,13,16].

In our previous works, we have substantiated the composition and method for preparation of compressible MCGs with a combination of lysozyme hydrochloride (LH) and ascorbic acid (AsA), which are intended for the prevention and treatment of dental diseases, in particular inflammatory diseases of the periodontium (gingivitis, periodontitis), mucous membranes (stomatitis), caries and manifestations of xerostomia [17,18,19,20]. The present study focuses on the mechanical, textural, release, and chewing perception characteristics of MCGs with the mentioned active pharmaceutical ingredients (APIs) depending on the compression force applied in the manufacturing process.

## 2. Materials and Methods

### 2.1. Materials

APIs and excipients used to obtain MCGs with a specification of their amount per gum, function in the formulation, and manufacturer are listed in Table 1.

All the chemicals and reagents used in the assay test were of analytical grade.

A commercially available functional chewing gum “XD Extra Drive AntiStress Relax” produced by Vitale-XD Ltd., Tallinn, Estonia, was used as a reference sample in texture profile analysis.

### 2.2. Preparation of Medicated Chewing Gums

The pre-compression stage of MCG preparation consisted of two steps. In the first one, granules were obtained from a mixture of LH, powder taste additive “Apple” and intense sweetener sucralose. For this purpose, powders were dry-blended, wetted with ethyl alcohol 96% and then granulated through a sieve and dried at room temperature. The second step included blending the granules with other ingredients: AsA, the chewing base Health in Gum^®^ PWD 01 (HiG PWD 01) and Syloid^®^ FP244. To achieve the homogeneous distribution of Apple liquid flavor in the mass for compression, it was sprayed onto the Syloid^®^ FP244 adsorbent and thoroughly mixed until a dry mixture was obtained. Magnesium stearate was added at the final stage of blending before compression to prevent sticking of MCGs to the surface of the punches during the tableting process [17,18,19].

Round flat-faced gums with a mass of 1000 mg and a diameter of 13 mm were produced on a laboratory single-punch tablet machine (model HTM-01E, Mariupol Plant of Technological Equipment, Mariupol, Ukraine) equipped with a force-measuring tool. Four MCG batches compressed at different force values, i.e., 5, 7, 10, and 15 kN (100 dosage units in each one), were obtained. The values of 5, 10, and 15 kN compression force was chosen based on similar formulation studies [12,21,22,23], and 7 kN—taking into account the recommendation of HiG^®^ manufacturer for production of compressible MCGs [12].

### 2.3. Evaluation of Prepared MCGs

The prepared gums were evaluated by their geometrical parameters, mechanical resistance, surface and internal structure characteristics, texture profile, release behavior, and perception attributes during mastication.

#### 2.3.1. Geometrical Parameters of MCGs

The diameter and thickness of MCGs were measured using digital micron caliper IP67 (Microtech, Kharkiv, Ukraine). In total, 10 dosage units were used for determination.

#### 2.3.2. Mechanical Resistance of MCGs

Mechanical resistance of MCGs was assessed by two pharmacopoeial tests, which are “Resistance to crushing of tablets” (Ph.Eur. 9.0, Chapter 2.9.8) and “Friability of uncoated tablets” (Ph.Eur. 9.0, Chapter 2.9.7) using a Monsanto hardness tester (Campbell Elec., Mumbai, India) and the PTF 20E friability apparatus (Pharma Test, Hainburg, Germany), respectively. To perform the “Friability” test, 10 dosage units were used, and drum rotation parameters were set to be (25 ± 1) rpm for around 4 min (required for 100 rotation times).

#### 2.3.3. Surface and Internal Structure Characterization

MCGs compressed at different compression forces were evaluated using a digital microscope Andonstar ADSM301 (Shenzhen Andonstar Technology Co., Ltd., Shenzhen, China) in regard to their surface smoothness and character of particle arrangement (i.e., compactness) inside the gums.

#### 2.3.4. Texture Profile Analysis

The texture properties of MCGs were investigated by penetration test using a texture analyzer TA.XT.plus (Stable Micro Systems Ltd., Godalming, Surrey, UK) using as a reference sample a commercially available compressed functional chewing gum “XD Extra Drive AntiStress Relax”. The penetration test evaluates the deformation response of the product to a stainless steel needle probe P/2N (2 mm thickness). To carry out the test, the gum was placed by its center under a needle probe, which then was set to penetrate the sample at a constant load of 5 kg and a speed of 2 mm/s to a depth of 3 mm. Two main parameters were registered: hardness—the maximum force value required for a probe to overcome mechanical resistance of the product while reaching penetration target; and adhesiveness—the force value required for a probe to overcome attractive forces (sticking) between its surface and the surface of the product being investigated. The analysis was performed at room temperature (25 ± 2) °C.

#### 2.3.5. In Vitro Drug Release Study

The drug release from MCGs was studied by the dissolution method according to pharmacopoeial test “Dissolution test for medicated chewing gums” using a chewing apparatus B (Ph.Eur. 9.0, chapter 2.9.25), which simulates the process of chewing (Erweka GmbH, Langen, Germany). The dissolution parameters were set as follows: number of chews per min—60; the distance between the chewing surfaces—1.4 mm; the dissolution medium—20.0 mL of phosphate buffer R2 solution (pH 6.0); sampling time points—5, 10, 15, 20, 30 min; sampling volume—2.0 mL (each sampling was replaced by 2.0 mL of pre-heated phosphate buffer R2 solution). The dissolution test was carried out on six MCGs for each batch.

The LH assay was carried out spectrophotometrically using a Specord 200 spectrophotometer (Analytik Jena AG, Jena, Austria) as described in the Japanese Pharmacopoeia (JP XVII) monograph for LH [24]. In total, 1 mL of aliquot was placed into a volumetric flask and adjusted by phosphate buffer (pH 6.2) to approximate LH concentration of 0.01 mg/mL after that 10 mL of Micrococcus luteus suspension in the same diluent (which absorbance at 640 nm is about 0.65) was added. The absorbance was determined at 640 nm using two standard solutions with a concentration of Lysozyme JP Reference Standard (PMRJ, Japan) of 0.01 and 0.005 mg/mL.

AsA assay was performed by redox-based iodatometric titration according to Ph.Eur. monograph 01/2011:0253 assay methods [14]. To 1 mL of aliquot, 10 mL of 0.1 M hydrochloric acid, 0.5 mL of a freshly prepared 10 g/L potassium iodide solution, and 2 mL of starch solution were added. The resulting solution was titrated with 0.0167 M potassium iodate until a violet-blue color was obtained.

#### 2.3.6. Chewing Perception Assessment

Six volunteers (3 females and 3 males, aged between 22 and 45 years) assessed four batches of MCGs compressed at different pressures. All of the study participants had a dentition with at least 16 natural elements and considered themselves in good health. The volunteers were asked just to chew the sample of each MCG batch for 20 min. Then, they had to give their scores from 1 to 5 points for such criteria as “tactile smoothness”, “initial hardness”, “crumbliness”, “rate of integral gum mass formation”, and “sticking to teeth”, where 1 corresponds to the lowest and 5 to the highest level of intensity for all criteria.

### 2.4. Statistical Analysis

Statistical analysis was performed using Microsoft Office Excel 2016. All experimental determinations were done in triplicate, and results are presented as mean value ± standard error (SE) or standard deviation (SD). Significant levels were defined at *p* < 0.05.

## 3. Results and Discussion

The data on geometrical parameters and mechanical resistance characteristics of MCGs produced at different compression force values are presented in Table 2.

**Figure 1:**
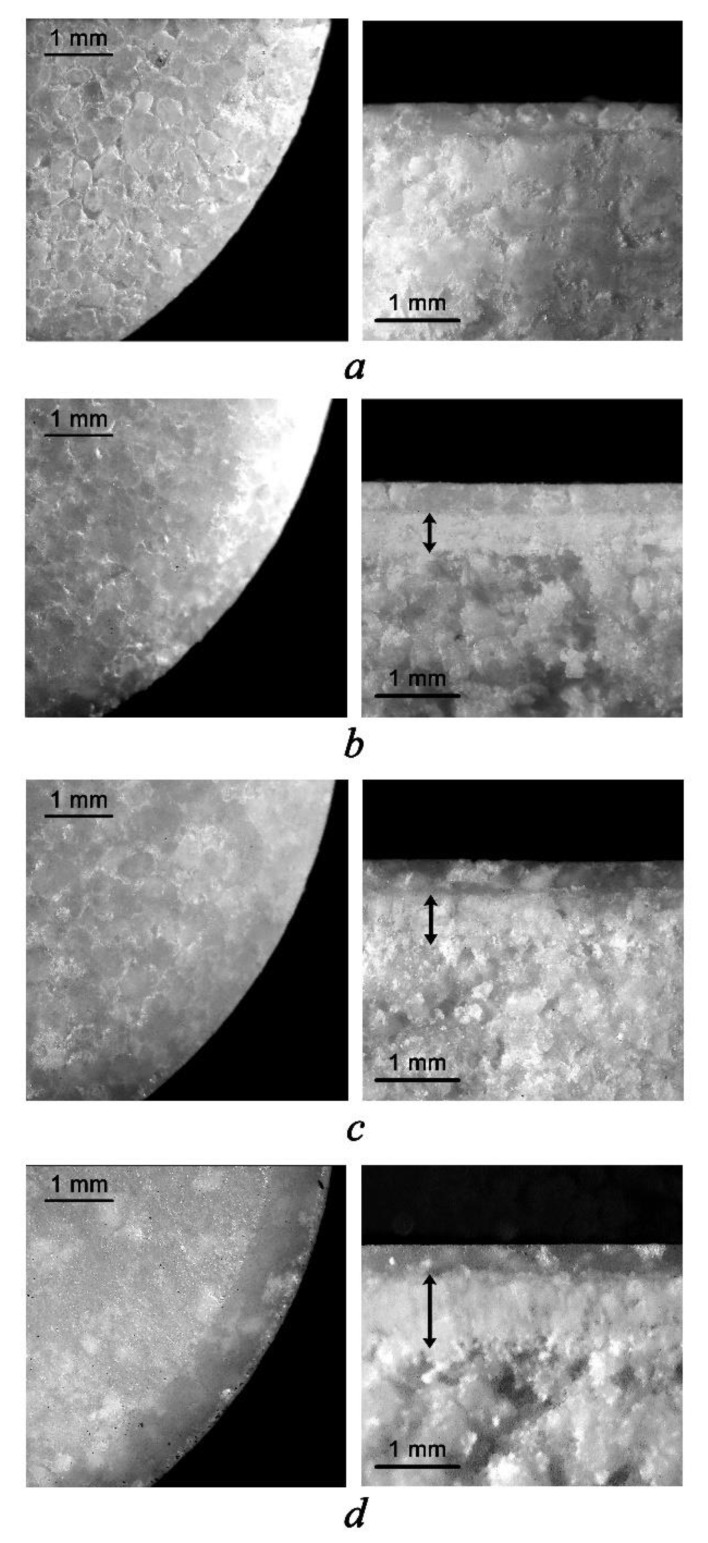
Digital microscopic images (20-fold magnification) of the surface and vertical cross-section of MCG batches compressed at different compression force values: (**a**) 5 kN; (**b**) 7 kN; (**c**) 10 kN; (**d**) 15 kN.

According to Table 2, parameters of MCGs such as thickness and friability were insignificantly affected by compression force, whereas increasing pressure during the manufacturing process resulted in statistically significant enhancement of MCG resistance to crushing. It should be noted that resistance to the crushing test did not lead to any break of gums, but only deformation, which is obviously related to the plastic and elastic nature of the chewing gum base. Small values of friability also can be addressed to the same property of MCGs, although there was some tendency of decreasing friability with raising the compression force. Two main mechanisms are suggested to be responsible for tablet friability—edge chipping and surface abrasion. The first is linked to tablet microstructure, i.e., porosity, and the second occurs when particles randomly detach from the tablet surface [25]. That is why a detailed inspection of MCG surface character and internal particle arrangement by using digital microscopy was carried out (Figure 1).

Microscopic investigation revealed that applying different compression force values resulted in an apparent distinction of surface character between four batches of MCGs (although the differences in MCG surface were not observable without magnification). As can be seen from the photos (Figure 1), increasing the compression force led to smoothing the MCG surface and reducing its relief. The latter appears to be formed by particle edges, which were still observed if the pressure was not enough to flatten individual particles into a single surface layer. Most clearly this was seen in the case of MCGs compressed at 5 kN, whereas for the gums obtained at 7 kN and then 10 kN, surface relief becomes less and less evident while it practically disappears for 15 kN batch gums.

The distinctions of MCGs compressed at different compression force values were also noticeable in their vertical cross-section views. For all batches of MCGs, except the one compressed at the lowest force, three layers of internal particle arrangement can be seen. The upper layer consists of the particles directly contacting with the punch of the tablet machine. This layer has the most structural unity since the particles forming it undergo such deformation that they just merge with each other. It should also be noticed that the thickness of the upper layer stayed practically the same in all four batches of MCGs, despite increasing pressure. However, there was a prominent effect of compression force on the following layer thickness, which is intermediate between the upper layer and the last one. While the last layer represents more chaotic packing of the particles (and so, it is most porous and not so compacted), the intermediate layer demonstrates a very close arrangement of the particles and although the individual particles are distinguishable, the space between them, i.e., porosity, is extremely low. This layer was hardly detected in MCGs compressed at 5 kN; however, with increasing compression force, the thickness of the intermediate layer (shown as a black arrow in Figure 1) also increases.

**Figure 2:**
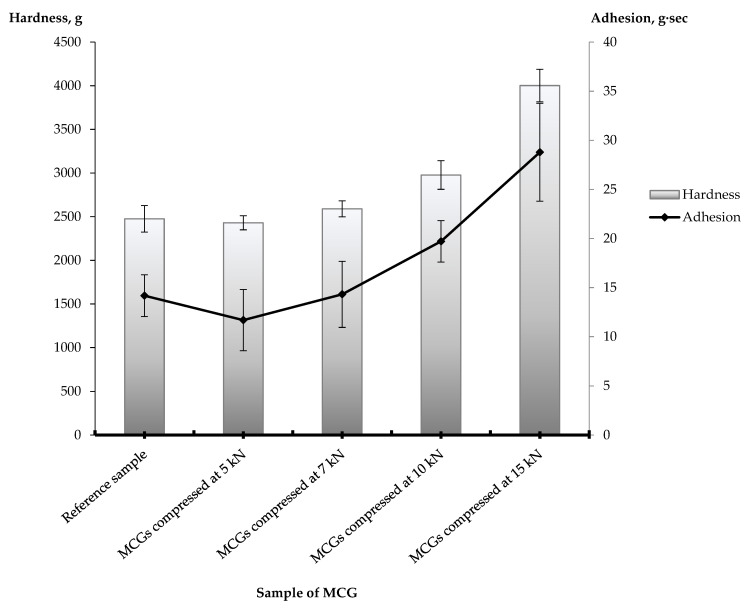
Comparative textural profile analysis of MCGs with LH and AsA compressed at different compression force values and a functional chewing gum “XD Extra Drive AntiStress Relax” (the values are mean ± SD, n = 3, *p* < 0.05).

The unbreakable behavior of MCGs during the resistance to crushing test actually reflects the main feature of this dosage form, as it is intended for chewing and thus must undergo plastic and elastic deformation, providing a relatively long mastication process. Additionally, contrary to common tablets, the hardness of MCGs is not so much important for mechanical stability (e.g., during transportation), but rather for chewiness—the extent of deformation caused by a certain load applying to the gum. For objective evaluation of product chewiness, a texture profile analysis is often used. Figure 2 shows the results of a texture profile analysis carried out for MCGs with LH and AsA compressed at different compression force values and functional chewing gum “XD Extra Drive AntiStress Relax” as a reference sample.

According to the hardness and adhesion values, the MCG batches are as follows: 15 kN > 10 kN > 7 kN > 5 kN. That is, the more compression force was applied, the harder and more adhesive were the gums obtained. This can be explained by the following considerations. During the decompression phase, the particles of MCG ingredients, especially the gum base, elastically and plastically deform and/or fracture. The larger compaction forces increase the solid fraction contact area. The adhesive mechanisms will produce larger adhesive forces as the contact area increases. In addition, the heat generated during powder compaction may partially melt particles, resulting in a further increase of the adhesive properties [26]. Therefore, the adhesive force increases with increasing compression force. That is why an unreasonably high compression force may eventually lead to difficulty in chewing MCGs. Consequently, this will contribute to the impairment of the product’s consumer properties. At the same time, the comparison with commercially available compressed chewing gum “XD Extra Drive AntiStress Relax” revealed the most textural similarity of the reference sample to MCGs obtained at 5 and 7 kN.

**Figure 3:**
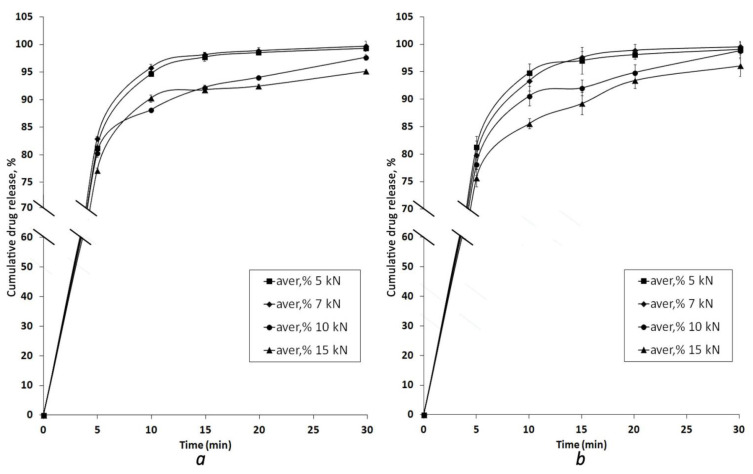
Cumulative release of LH (**a**) and AsA (**b**) from four batches of MCGs compressed at different compression forces (values are expressed as mean ± SD, n = 6, *p* < 0.05).

One of the biopharmaceutical parameters for traditional tablets, which can be greatly affected by the increasing hardness of the product, is the dissolution profile [27]. This dependence is more crucial for low water-soluble drugs and not so obvious for highly water-soluble ones. Compared with common tablets, as was already mentioned above, MCGs have the advantage of chewing as an enhancing tool in the release of APIs. So, even in the case of well water-soluble drugs (for instance, LH and AsA), the driving mechanism of API release from MCGs into the salivary fluid of the oral cavity is not only diffusion but primarily mechanical stimulus. In this regard, the kinetics of drug release from four batches of MCGs with LH and AsA compressed at different compression force values were studied (Figure 3 and Table 3).

From the release kinetic plots given in Figure 3 and the data presented in Table 3, it can be seen that nearly 80% of both LH and AsA were detected in the dissolution medium after 5 min of the test for all MCG batches being studied. However, the magnitude of the compression force had some effect on the dissolution profile. In a general way, MCGs compressed at a higher force showed slower release, though the most difference was observed at the first two sampling points and less pronounced at the end of the dissolution test. This may indicate that initial drug release, to a great extent, is driven by mechanical treatment via the chewing process, and the harder an MCG, the less effectively it can be treated, i.e., chewed. Further drug release becomes more and more diffusion-mediated and APIs leave the gum matrix in a manner not associated with its initial hardness. The effect of compression force is especially evident comparing release kinetic curves of the highest and the lowest compression force batches, whereas 5 kN and 7 kN gums show practically the same release behavior, e.g., average release percentages of AsA for 5, 7, 10, and 15 kN MCGs batches at 10 min were 94.87, 93.34, 90.62, and 85.58%, respectively, while at 30 min they were 99.08, 99.55, 98.88, and 96.11%, respectively. Nonetheless, considering that recommended duration of mastication of dental chewing gums is around 20 min [28,29], MCGs compressed at lower compression force values would provide a more complete release of APIs during this time.

So, according to the results described above, lower compression forces seem to be more preferable from the point of view of better textural and release profiles of the gums obtained. This assumption is also supported by the fact that all MCGs including those produced at lower compression forces completely met the regulatory requirements (Ph.Eur.) of their resistance to mechanical stress. However, there is one point indicating otherwise—some imperfections of surface and internal particle arrangement detected by digital microscopy for MCGs compressed at lower compression forces. Obviously, these imperfections potentially can affect mouthfeel during the chewing process and, in this case, impair the acceptability of the preparation by the patient. Therefore, evaluation of mouthfeel during chewing experimental MCG batches was performed involving six volunteers.

**Figure 4:**
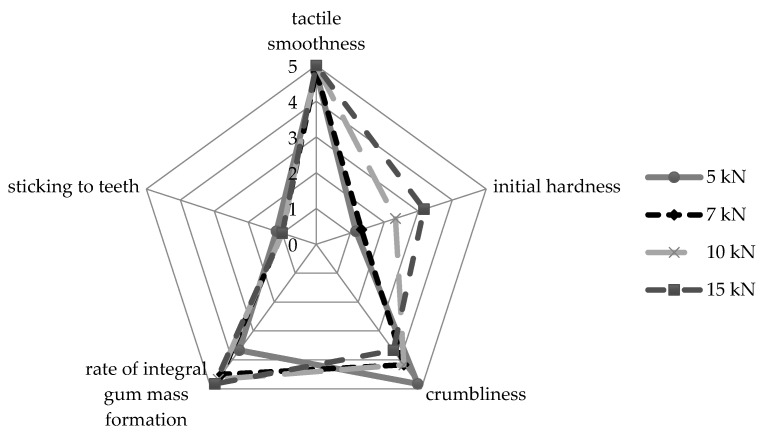
Diagram of average chewing perception scores for MCG batches compressed at different compression forces.

Earlier it was described that when chewing the gum obtained by the compression method, it initially crumbles into many particles, which, after wetting by saliva, come together to form an integral gum [30]. Based on this, as well as the authors’ personal experiences, the following criteria were chosen for mouthfeel assessment during chewing: “tactile smoothness”, “initial hardness”, “crumbliness”, “rate of integral gum mass formation”, and “sticking to teeth”. The results of average scores on each of the mentioned criteria for all experimental batches of MCGs are depicted as a diagram given in Figure 4.

According to average scores, change of compression force applied during the production process does not lead to significant differences in such criteria as “tactile smoothness” and “sticking to teeth”. All samples of MCGs were perceived as smooth (average scores ranged from 4.83 ± 0.37 both for 5 and 7 kN to 5.00 ± 0.00 for the other batches) and not sticking to teeth (from 1.00 ± 0.00 for 7, 10, 15 kN to 1.17 ± 0.37 for 5 kN batch). This indicates that differences that are registered by in vitro methods described above (i.e., digital microscopy characterization of MCG surface or adhesion value determination by texture profile analysis) can hardly be distinguished in vivo. On the other hand, the most perceptible differences between batches of MCGs are found to be in initial hardness, crumbliness, and rate of integral gum mass formation. The lower the compression force used is, the less hard the gum is at initial biting (average scores were 1.17 ± 0.37, 1.33 ± 0.47, 2.33 ± 0.75, and 3.17 ± 0.90 for 5, 7, 10, and 15 kN batches, respectively), the more it crumbles (4.83 ± 0.37, 4.17 ± 0.69, 4.17 ± 0.37 and 3.67 ± 0.47 for 5, 7, 10 and 15 kN batches, respectively) and the slower (at least, subjectively) its particles merge with each other under chewing into one integral gum mass (3.67 ± 0.75, 4.50 ± 0.76, 4.67 ± 0.47 and 4.83 ± 0.37 for 5, 7, 10 and 15 kN batches, respectively).

The score ranges for “initial hardness”, “crumbliness” and “rate of integral gum mass formation” can be completely explained by digital microscopy results. Obviously, the formation of the intermediate layer in the microstructure of MCGs (described above) serves as a pre-step for merging MCG particles into one mass under chewing; however, this layer is associated with increasing effort to crumble at an initial bite. In the general case, less crumbliness is more preferable, but along with this, the initial hardness of MCGs rises, which may have a more negative effect on the acceptability, considering a possibility of painful chewing in patients suffering from periodontal diseases [31,32]. So, this issue requires certain compromise and, in the authors’ opinions, the best solution is a compression force value of 7 kN, also recommended by the gum base manufacturer Cafosa. As shown in this study, applying a compression force of 7 kN allows to achieve lower crumbliness as compared to 5 kN, but practically does not affect the initial hardness and release behavior of the MCGs obtained.

It should be noted that the findings of this study correlate with previous reports devoted to the same problem—the effect of compression force on the properties of MCGs obtained. Jójárt I. et al. concluded that lower compression force was more suitable from the aspect of the chewiness of the gums obtained. A value of 5 kN was substantiated to be the best among others, such as 10, 12.5, and 15 kN [23]. In another work, it was also demonstrated that the type of magnesium stearate and the compression force value do not affect the release of the API from MCGs. On the other hand, neither scanning electron microscopy (SEM) nor porosity measurement revealed any differences in the internal microstructure of MCGs compressed at 5 and 10 kN: both apparent density and true density of the compressed gums did not differ significantly, and each of the samples had the same high porosity [21,22]. In our opinion, some discrepancies between the results presented herein and in earlier studies may be explained by the methods used. For instance, as compared to nanoscale images, microscale ones may occur more informative for internal particle arrangement characterization. Another limitation may be related to the parameters of compression equipment used in the studies. Besides the value of compression force, the time of applying it, i.e., so-called dwell time, as well as the size of a die and punches also matter [33]. Nevertheless, the novelty of the present study consists of linking and explanation of the textural, in vitro release and chewing perception properties of MCGs produced at different compression forces from the point of view of their internal particle arrangement. The authors strongly believe that assessing particle arrangement as well as chewing perception described herein can be a useful approach to alleviate possible discrepancies related to compression equipment parameters in similar investigations.

## 4. Conclusions

All batches of MCGs compressed at 5, 7, 10, and 15 kN compression force values completely met the requirements of Ph.Eur. on resistance to crushing and friability tests; however, change in the compression force resulted in differences in textural, release, and chewing perception properties of the samples. The higher compression force led to harder gums, characterized by the less efficient release of APIs at the initial bite, but also less crumbliness and faster merging into one gum mass during chewing. These differences can be explained by a certain internal particle arrangement and, in particular, the formation of a layer where particles are very closely compacted. By the means of digital microscopy, it was found that the thickness of such layer directly depends on the compression force applied. Despite less crumbliness appearing to be more comfortable, it is associated with increased effort at initial biting, which may be not acceptable for patients suffering from painful chewing caused by periodontal tissues inflammation. Thus, among compression forces of 5, 7, 10, and 15 kN, a value of 7 kN was chosen since it allowed to achieve lower crumbliness as compared to 5 kN, but practically did not affect the initial hardness and release behavior of MCGs obtained.

## Figures and Tables

**Table 1:** APIs and excipients used to obtain MCGs.

Name of Ingredient	Amount, mg/per Gum	Function in the MCG Formulation	Manufacturer
Lysozyme hydrochloride	10.0	API	Bouwhuis Enthoven B.V., Raalte, The Netherlands
Ascorbic acid	20.0	API	Foodchem International Corporation, Shanghai, China
Solo Sucralose Non-Micronised NF	1.5	Intense sweetener	V.B. Medicare Pvt. Ltd., Hosur, India
Nat Apple Flavor Wonf	20.0	Taste additive	Kerry Inc., Kuala Lumpur, Malaysia
Apple FLV LQD FA-BO2980	6.0	Flavor	Kerry Inc., Kuala Lumpur, Malaysia
Syloid^®^ 244FP	10.0	Moisture scavenger, carrier for liquid flavor, glidant	Grace GmbH & Co. KG, Worms, Germany
Magnesium stearate	15.0	Lubricant	S.D. Fine Chemicals Ltd., Mumbai, India
Health in Gum^®^ PWD 01	Up to 1000.0	Chewing gum base	Cafosa Gum SA, Barcelona, Spain

**Table 2:** Geometrical parameters and mechanical resistance characteristics of MCGs.

Compression Force Value, kN	Geometrical Parameters, mm		Resistance to Crushing, N	Friability, %
	Diameter	Thickness		
5	13.02 ± 0.01	5.62 ± 0.03	71 ± 2	0.121 ± 0.001
7	13.01 ± 0.01	5.50 ± 0.02	78 ± 2	0.102 ± 0.001
10	13.01 ± 0.01	5.44 ± 0.05	80 ± 1	0.090 ± 0.001
15	13.00 ± 0.01	5.21 ± 0.03	92 ± 2	0.054 ± 0.001

Note. Measurement procedures were repeated three times using 10 dosage units for each testing parameter. The values are given as mean ± SD, *p* < 0.05. Compression force value was kept constant for each batch.

**Table 3:** Kinetics of APIs release depending on time.

Compression Force Value, kN	API	Released Amount of APIs, %				
		Sampling Time, min				
		5	10	15	20	30
5	AsA	81.35 ± 1.90	94.87 ± 1.56	97.05 ± 2.45	98.12 ± 0.82	99.08 ± 1.47
	LH	81.25 ± 0.38	94.68 ± 0.63	97.75 ± 0.48	98.55 ± 0.57	99.34 ± 0.91
7	AsA	79.86 ± 2.53	93.34 ± 1.80	97.65 ± 1.08	98.93 ± 1.07	99.55 ± 0.52
	LH	82.95 ± 0.56	95.83 ± 0.45	98.17 ± 0.78	98.90 ± 0.45	99.71 ± 0.59
10	AsA	78.19 ± 2.02	90.62 ± 1.77	92.11 ± 1.41	94.90 ± 1.43	98.88 ± 1.52
	LH	80.29 ± 0.69	88.12 ± 0.31	92.21 ± 0.47	94.02 ± 0.37	97.67 ± 0.56
15	AsA	75.66 ± 1.55	85.58 ± 0.93	89.25 ± 2.05	93.45 ± 1.48	96.11 ± 1.95
	LH	77.13 ± 0.52	90.25 ± 0.66	91.77 ± 0.48	92.45 ± 0.34	95.11 ± 0.36

Note. Measurement procedures were repeated three times using 6 dosage units. The values are given as mean ± SD, *p* < 0.05.

## Data Availability

All data are available upon request.
